# The quest for a mechanistic understanding of biodiversity–ecosystem services relationships

**DOI:** 10.1098/rspb.2015.1348

**Published:** 2015-10-22

**Authors:** Clare Duncan, Julian R. Thompson, Nathalie Pettorelli

**Affiliations:** 1 Institute of Zoology, Zoological Society of London, Regent's Park, London NW1 4RY, UK; 2 Department of Geography, University College London, Gower Street, London WC1E 6BT, UK

**Keywords:** biodiversity, ecosystem services, ecosystem function, mechanisms, proxies, biodiversity–ecosystem services relationships

## Abstract

Ecosystem services (ES) approaches to biodiversity conservation are currently high on the ecological research and policy agendas. However, despite a wealth of studies into biodiversity's role in maintaining ES (B–ES relationships) across landscapes, we still lack generalities in the nature and strengths of these linkages. Reasons for this are manifold, but can largely be attributed to (i) a lack of adherence to definitions and thus a confusion between final ES and the ecosystem functions (EFs) underpinning them, (ii) a focus on uninformative biodiversity indices and singular hypotheses and (iii) top-down analyses across large spatial scales and overlooking of context-dependency. The biodiversity–ecosystem functioning (B–EF) field provides an alternate context for examining biodiversity's mechanistic role in shaping ES, focusing on species' characteristics that may drive EFs via multiple mechanisms across contexts. Despite acknowledgements of a need for B–ES research to look towards underlying B–EF linkages, the connections between these areas of research remains weak. With this review, we pull together recent B–EF findings to identify key areas for future developments in B–ES research. We highlight a means by which B–ES research may begin to identify how and when multiple underlying B–EF relationships may scale to final ES delivery and trade-offs.

## 1. Introduction

In recent decades, conservation science has seen a gradual shift of focus away from traditional ‘fortress conservation’ towards balancing the requirements of both biodiversity and humans [1]. The fundamental means by which people benefit from the world's ecosystems is through the goods and services that their healthy functioning provides [2]. By directly producing goods and facilitating ecosystem functions (EFs; [3,4]), biodiversity may be a key driver of ecosystem services (ES) [5,6]. Areas of high importance for biodiversity conservation and ES delivery can sometimes be identified [7,8], meaning that there may be clear co-management opportunities [9,10]. This has led to increasing policy-level emphasis on whole-ecosystem approaches to biodiversity conservation [11]. However, significant debate remains over the relevance of ES approaches to biodiversity conservation [12,13], especially so as our understanding of biodiversity and ES linkages (B–ES relationships) remains incomplete [5]. A linear positive association between biodiversity and delivery of individual ES is indeed not always manifest. B–ES relationships have been found to (i) take varying forms and shapes (e.g. nonlinear relationships; [14–16]), (ii) display mixed relationships [5] or (iii) be altogether non-existent [5,6]. The existence of mixed B–ES relationships for individual ES highlights the great variability in the influence of biodiversity on a given ES in a given context [5,6]. Moreover, variation in individual B–ES relationships can ultimately result in trade-offs, as well as synergies, between multiple ES [5,15–20].

So why do we see such variability in B–ES relationships? B–ES research has historically taken a rather top-down, correlative approach, with the underlying ecological mechanisms being mostly ignored [18,21]. As a result, and despite the ever-increasing body of the B–ES literature, we are still a long way from understanding the mechanisms shaping B–ES relationships across landscapes. Some of the means by which we may begin to deepen our understanding of biodiversity's mechanistic influence on ES lie within the theory, recent findings and methodologies of the biodiversity–ecosystem functioning (B–EF) literature. However, the B–ES and B–EF fields are increasingly acknowledged to be detached [5], rarely working together and generally being conducted in different contexts and at completely different scales. Lessons from the wealth of emerging mechanistic B–EF research are not currently extended to examining B–ES relationships. We may thus be looking at too simplistic a picture of B–ES relationships and overlooking opportunities to link biodiversity and ES delivery via full functional pathways.

This review aims to bridge this gap, drawing from recent findings and theories from the B–EF literature to develop a greater mechanistic understanding of B–ES relationships, working from the bottom-up and extending linkages between biodiversity and the EFs underlying individual ES to multiple ES delivery across landscapes. We begin by highlighting key areas for concern in current understanding of B–ES; we then discuss lessons to be learned from the B–EF field; we finally introduce a hierarchical research framework based on combining recent theoretical advances in both fields to enhance the mechanistic basis of current B–ES understanding.

## 2. The role of biodiversity in ES delivery: key areas for concern

### (a) Inappropriate indices and proxies

#### (i) ES

**Figure 1.**
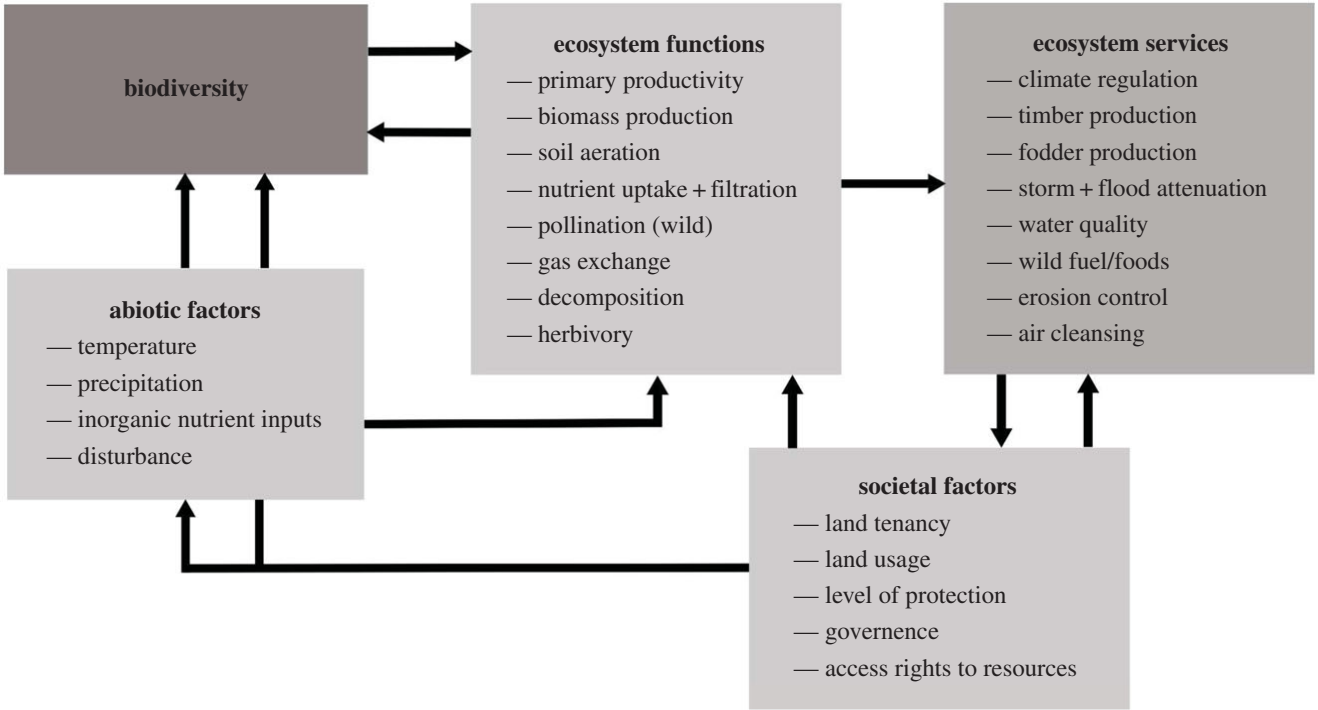
Schematic of the complex linkages (‘cascade’ [25]) involved in final ES delivery. The examples of ES, underlying ecosystem functions (EFs; also termed ‘intermediate services’ [24]), abiotic and societal factors represent a non-exhaustive selection.

The B–ES field has historically seen much ambiguity in ES definition [4,22–24]. Rigorous re-characterization has now resulted in clear separation of ‘intermediate services' (in reality EFs and referred to as such hereafter) that underpin ‘final ES' delivery (figure 1; [24,25]). Observational B–ES research has, however, been slow to adopt this classification [4,24], the result being use of misinformed and inconsistent ES proxies [26–28]. Underlying EFs are routinely measured (e.g. soil retention, net primary productivity; NPP), under the assumption that such proxies will hold to single [8,29] or multiple final ES [30] (see also [27]). A recent study has revealed that this latter assumption is not always plausible, with some ES (pest control and pollination) diminished in highly productive cropland areas [31]. Use of partial EF proxies means that complete B–ES linkages are rarely explored, reducing the mechanistic and predictive capacity of B–ES research [21]. For example, ecosystem carbon sequestration and storage ES relies upon, e.g. plant biomass production, nutrient cycling, soil turnover and water retention EFs, and these may have complex interconnections and independent linkages with biodiversity (e.g. [29,32]). Furthermore, there has been much variability in the indices used to quantify final ES ([33]; i.e. the field has yet to reach consensus on a standard set of proxies [34]); this is currently limiting our ability to generalize from observed B–ES relationships.

#### (ii) Biodiversity

Inherent to the Convention on Biological Diversity's definition is that biodiversity is multifaceted and a complex beast to measure (box 1; [35]). Strict B–ES research relies on simplistic, species-level indices of biodiversity, e.g. species richness (box 1; [6,27]; but see [43]), despite acknowledgements of their limited relevance [30]. There is indeed little theoretical basis that increasing units of species should always result in increased ES delivery [3]. Biodiversity is fundamentally composed of three axes (taxonomic, structural (community complexity) and functional diversity), and species richness captures little of this overall information [35]. Species identity and relative abundance may instead assert key controls on EFs and final ES [44–46]. Use of simplistic indices means B–ES research currently lacks evaluation of *how* organisms contribute to final ES delivery.

Box 1. **Biodiversity: definitions and selected taxonomic and functional indices.**

**CBD definition of biodiversity**

The variability among living organisms from all sources including, inter alia, terrestrial, marine and other aquatic ecosystems and the ecological complexes of which they are part; this includes diversity within species, between species and of ecosystems.

**Taxonomic diversity**

*Species richness*. The number of species in a community or taxonomic group in a specific area (Hill Numbers of ‘True Diversity’ *N*_0_; [36]).
*Alpha diversity.* Combined diversity measures describing species richness and evenness of a community (Hill Numbers ^1^*N*, ^2^*N* … ^Inf^*N*; [36]).
*Species evenness*. The relative abundance structure of species in a community (Hill Numbers evenness = ^2^*N*/^1^*N*; [36]).
*Gamma diversity*. The total diversity of species across a landscape (e.g. Hill Numbers ^1^*N_*γ*_* = ^1^*N_*α*_* × ^1^*N_*β*_*, ^2^*N_*γ*_* = ^2^*N_*α*_* + ^2^*N_*β*_*; [36,37]).
*Beta diversity*. The diversity of species among communities; the difference in composition and diversity between communities occupying different areas across a landscape (e.g. Hill Numbers 1*N_*β*_* = ^1^*N_*γ*_*/^1^*N_*α*_*, ^2^*N_*β*_* = ^2^*N_*γ*_* − ^2^*N_*α*_*; [36,37]).

**Functional diversity**

Functional diversity comprises three major components: richness, evenness and divergence [38].

*Functional richness*. A measure of the functional (niche) space filled by a community, e.g. single-trait: FRci [38]; multi-trait: FRic [39].
*Functional evenness*. A measure of the regularity of functional trait distribution in trait space according to abundances, e.g. single-trait: Evar [40]; multi-trait: FEve [39].
*Functional divergence*. A measure of variance in functional traits in trait space, maximized when the most abundant species are highly divergent, e.g. single-trait: FDvar [38]; multi-trait: FDiv [39].
*Functional dispersion*. An index combining functional richness and functional divergence, e.g. single- or multi-trait: Rao's Q [41]; multi-trait: FDis [42].
*Community-weighted mean functional traits*. A measure of dominant functional traits; the mean functional trait value for a given trait within a community, weighted by abundance.

B–ES research has frequently indexed biodiversity using policy- over ecologically-relevant measures: threatened species richness [47,48] or biodiversity priority areas [15]. While clearly important in identifying existing spatial congruence in conservation priorities, findings from such studies serve only to describe spatial patterns in incomplete B–ES linkages. The ecological mechanisms driving B–ES relationships are controlled by the whole suite of organisms in a given area [43,49–51]. Excepting for some specific ES (e.g. pollination; [14,52,53]), B–ES studies have then focused on groups unlikely to produce a direct mechanistic influence, linking e.g. diversity of mammals to carbon storage or trees to game production [16,48]. EF is controlled by intricate trophic interactions, yet much B–ES research ignores this inherent complexity (but see [49,54,55]).

### (b) The importance of scale

B–ES research has largely involved spatially correlative studies across extremely large management-relevant scales (e.g. [15,30,47,48]). Such studies have generated a wealth of knowledge on broad B–ES linkages and ES valuation [5]. However, working at such extensive spatial scales incurs substantial information loss regarding the mechanisms underpinning B–ES relationships, as key EFs promoted by organisms operate at much finer scales [14]. Common landcover-based B–ES studies [7,9], in addition to providing poor fits to actual ES data [19,26,56], do not enable examination of B–ES relationships within ecosystems. For example, while mangrove forest areal loss produces important trade-offs between coastal protection and shrimp farming [57], little is known about the role mangrove biodiversity itself plays in this relationship, despite it strongly influencing functionality [58]. Findings from B–ES relationships across multiple ecosystem types (but see [16,53]) may moreover be obscured by the type and diversity of ecosystems present. For example, carbon storage across the UK is greatest in areas of intermediate biodiversity, due to strong abiotic controls on carbon cycling in temperate uplands [29]. Use of coarse biodiversity data over large areas can then confound landscape-level biodiversity phenomena (box 1; [26]), weakening our understanding of local B–ES relationships by confusing spatial and temporal complementarity effects of beta and gamma diversity ([59]; box 1).

## 3. Lessons to be learned from B–EF research

### (a) Lesson 1: moving from species to functional traits

In contrast to B–ES research, the B–EF field has seen a greater focus on species' characteristics [3,44,46,51,60]. Increasing evidence now shows that the key means by which species influence EFs is through their functional traits (phenotypic attributes that direct niche exploitation; [44]), which may not always be well described by often-used measures of phylogenetic diversity [61]. While functional diversity (box 1) may theoretically increase with species richness in some contexts [3], taxonomic biodiversity measures (particularly species richness) have been found to explain little variance in EFs compared with functional trait indices [45].

### (b) Lesson 2: considering the existence of multiple mechanisms

**Figure 2.**
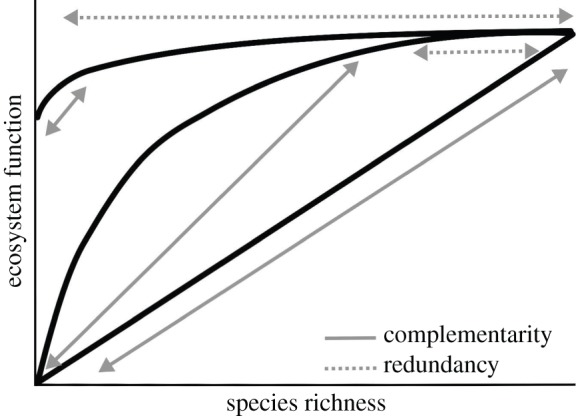
Representation of observed static species richness-based B–EF relationships. B–EF relationships can vary from linear to rapidly saturating, where high levels of ecosystem functioning occurs in the presence of few species [60,62]. Commonly observed saturating B–EF relationships show complementarity between species at low species richness (complementarity in niche partitioning resulting in increased overall resource use) driving increased functionality, while at higher levels of species richness many species may exhibit redundancy [60,62–64]. Note that static saturating curves do not imply actual functional redundancy in some species; temporal heterogeneity increases the insurance value of biodiversity through time [63–65].

In comparison to the B–ES field, which has focused almost exclusively on the hypothesis that biodiversity drives ES (but see [14,52,53]), B–EF research has explored multiple hypotheses for how organisms promote EFs: (i) the diversity hypothesis: mechanisms including niche complementarity and insurance (compensatory dynamics through space and time) (figure 2) and (ii) the mass ratio hypothesis (functional traits of dominant species chiefly promote EFs) [3,51,66,67]. Experimental B–EF research focusing on species richness has provided broad support for the diversity hypothesis [5,60,63,65–68]. Trait-based research has, however, shown that many EFs are driven predominantly by mass ratio (e.g. NPP, decomposition, nitrification, carbon content; [43,45,46,69–72]). Yet high functional diversity alongside dominant traits may additionally promote EFs [44,69,71,73,74]; especially so by providing stability [63,65]. Greater levels of biodiversity may be required to support multiple EFs simultaneously [63,68,75–77], as the functional traits and importance of complementarity may vary for different EFs ([45]; but see [51]). Given the high functional distinctiveness of rare species, biodiversity may thus remain paramount to maintaining multifunctionality in space and time [78].

### (c) Lesson 3: shifting from regional to within-ecosystem scales

In complete contrast to B–ES research, B–EF studies have routinely been conducted in controlled experimental settings at small scales [60]. Observational B–EF investigation is becoming increasingly common, and important studies now corroborate experimental findings within real systems (e.g. [79–82]; but see [83–85]). Data collection at small (i.e. plot-based) scales in both experimental and observational studies means B–EF research is conducted at meaningful extents across which biodiversity's linkages to EFs mechanistically operate, while simultaneously enabling comparisons across large areas (e.g. [80,81,84]). Small scales of data collection have importantly meant that B–EF research examines biodiversity's role in promoting EFs *within* specific ecosystem types (e.g. [80,81]). This focus on within-ecosystem type studies is crucial, as the nature of B–EF linkages, and the final ES they underpin (e.g. in converted versus natural systems; figure 1), can be highly context-dependent.

### (d) Lesson 4: relationships are context-dependent

In addition to biodiversity effects *per se*, EFs are driven by other interacting drivers: abiotic and climatic controls [44,63,80], disturbance [86,87] and management [88] (figure 1). The interplay between abiotic drivers, biodiversity and productivity is a key control on multifunctionality [30,44,64]. Both the number and identity of species promoting EFs differs according to the environmental context (e.g. CO_2_ and N concentrations; [63]), disturbance history [86] and ecosystem management [88], thus both the strength and form of B–EF relationships may vary strongly across contexts. Outside of experimental settings, B–EF relationships can be stronger because of a greater frequency of complementary species interactions [82,84,85]; or distinct dominance structures can enhance dominant species' influence in other systems [53]. It has been hypothesized that beyond the lower end of a species richness gradient, the main driver of EFs is community functional structure [70]. If the static influence of biodiversity on EFs can be captured by saturating positive curves [63,65,82], less productive, species-poor systems ([89,90]; e.g. deserts, mangroves) may display comparatively low redundancy, being consistently towards the left-hand side of these relationships (figure 2). However, while positive B–EF relationships have been observed in many species-poor systems (global drylands [81], boreal over temperate forests [80], early- over late-successional forests ([87]; but see [91])), very strong positive relationships have also been found in highly species-rich systems [84,85]. Furthermore, biodiversity remains the primary determinant of some EFs globally (e.g. decomposition; [92]).

## 4. Towards a more integrated, mechanistic understanding of B–ES relationships

We are beginning to acquire a good understanding of diversity and dominance-based functional B–EF relationships in given contexts [44,46,70]. However, substantial research is required if we are to gain a more mechanistic and predictive understanding of individual and multiple B–EF and B–ES relationships. Efforts must now be made to (i) quantify how multiple B–EF relationships scale up to final ES delivery and (ii) elucidate the pathway of EF-generated trade-offs between final ES across landscapes. Here we outline a step-by-step research framework through which these connections may begin to be explored.

### (a) Understanding final ES as a product of multiple EFs

An important redefinition of biodiversity's influence on ES has recently been outlined as a ‘multi-layered relationship’ [4]. Some final ES are delivered by organisms directly: a certain group of organisms acting as a good or carrying out a final ES (e.g. wild crop, fruit or game production, agricultural pollination) [4]. For such final ES, an important avenue of B–ES research explicitly links functional trait efficiency and abundance to ES delivery (‘ecosystem service providers’ [14,52]). However, biodiversity across multiple trophic levels facilitates many final regulating ES via the multitude of key EFs underpinning them (table 1; [4,27,43,64]). This full pathway of EF effects is not considered in B–ES research (but see [20,93]). ES research is unintentionally moving towards such a goal by examining ES ‘bundles’: identification of groups of ES commonly positively associated in space [15,19,94]. Synergies within ES bundles are a result of positive connections with similar underlying EFs. For example, the ‘forest services’ (carbon storage, timber, air cleansing, erosion control, recreation) and ‘soil and water services’ (water provision, soil carbon, infiltration) bundles across Europe [15] are positively underpinned by many similar EFs (table 1). However, we still lack quantitative understanding of interlinkages between multiple underlying EFs, and how separate biodiversity effects may mediate these interrelationships [20].

**Table 1.** Examples of commonly studied ES and the underlying EFs and main contributing trophic levels responsible for their delivery.

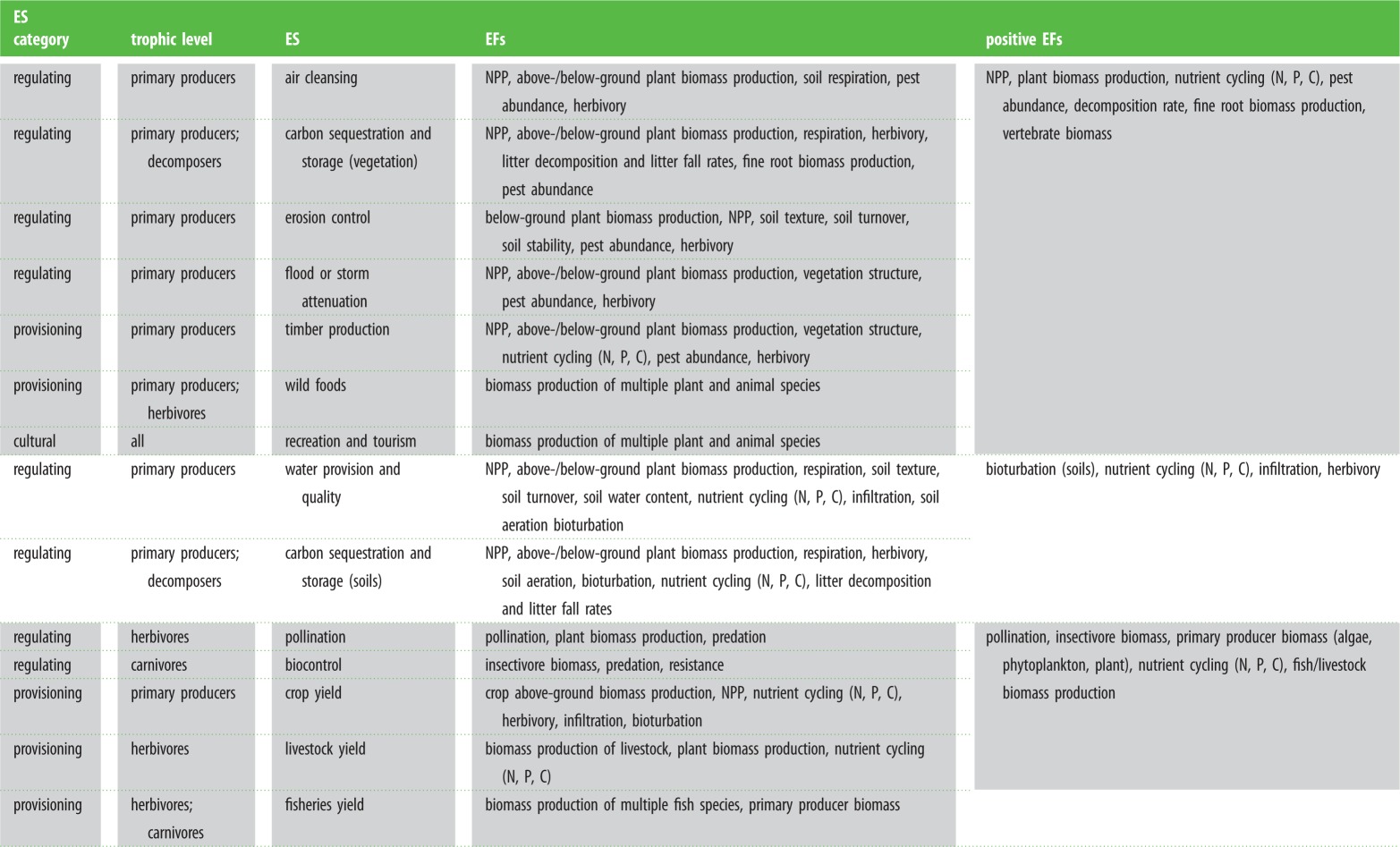

Looking to underpinning EFs is an essential next step for B–ES research if we are to ascertain biodiversity's mechanistic role. High levels of biodiversity may be required to drive those final ES underpinned by multiple EFs in a given context as (i) EFs positively promoting final ES are not always positively correlated [32,95], (ii) different EFs may contribute both positively and negatively [20,27], (iii) some final ES are the sum of contributions from multiple ecosystem compartments (e.g. total ecosystem carbon storage; [27]), (iv) different EFs are predominantly promoted by different groups of organisms (trophic or functional groups; table 1; [14]), and (v) the main mechanisms by which organisms promote different EFs may vary (diversity versus dominance; [44,45]).

### (b) Characterizing which EFs underpin ES delivery

A major future area for B–ES research lies in quantifying those key EFs contributing to final ES [5,44]. The B–EF field has seen much research into ecosystem multifunctionality, revealing the greater role of biodiversity in supporting multiple over single EFs [63,68,75–77,81]. Frameworks for quantifying ecosystem multifunctionality are fast-developing [96], and may enable exploration of B–EF linkages for groups of EFs underpinning specific final ES. However, inference from multifunctionality indices to identify key EFs underpinning final ES delivery is limited, especially so as multifunctionality B–EF linkages do not always reflect the strength, direction and mechanisms of all component individual B–EF relationships [97]. At the other end of the spectrum, ES research estimates ES via underlying EFs using ecological production functions [98]. However, these can range in nature from simplistic (carbon storage) to highly complex (water quality), ignore EF interlinkages and rely on basic linkages to biodiversity [93].

For quantification of key EFs, we here define the concept of ‘EF portfolios’ for given final ES, identifiable via plot-based or landscape-scale assessment of multiple sites. The process requires at each place simultaneously quantifying all measurable EFs potentially underlying a given final ES (table 1). This refers not only to positively contributing EFs but also to those potentially negatively impacting final ES delivery ([27]; e.g. herbivory for timber production, transpiration for water availability). It is then possible to assess (i) the average relative contribution of an EF and (ii) its ‘irreplaceability’ to final ES delivery (box 2). For the latter, threshold levels of final ES can be set, based on, e.g. stakeholder surveys or market value information [21], and EFs examined for their overall contribution to this threshold; i.e. do low values of one or few EFs consistently result in final ES delivery below the set threshold, or do other EFs make up the difference? Similarly to quantifying ‘ecosystem service providers’ [14], these two values of average relative contribution and ‘irreplaceability’ are summed to create an importance value for each EF. All EFs with high importance values are then considered within a given ES' EF portfolio (box 2). Importantly, EF portfolios can be determined by combining multiple proxy datasets, and may be central to identifying the relative utility of commonly used ES proxies [26].

Box 2. **A framework for establishing EF portfolios.**

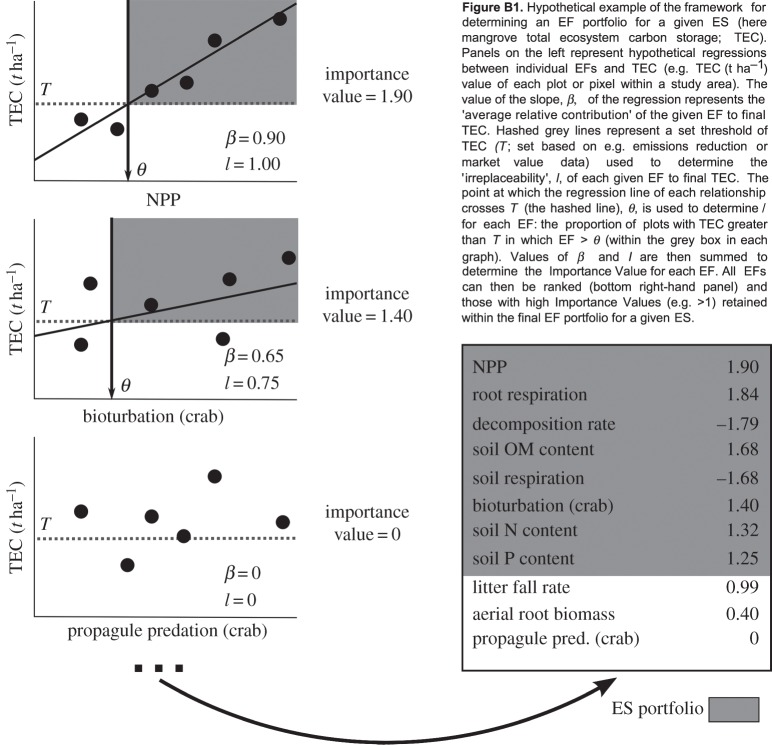

### (c) Quantifying synergies and trade-offs between EFs and final ES delivery

#### (i) Grouping EFs according to common traits and mechanisms

Can EFs be grouped according to the main groups of organisms and traits underpinning them in a given context? At least for some plant-mediated EFs key to underpinning some final ES (e.g. timber production, erosion control; table 1), the answer may be yes [20,45]. However, there may exist fundamental trait-based trade-offs between EFs [20], which may be severe if the main mechanism of their promotion is via dominant functional traits. Furthermore, diversity in some traits may positively influence EFs alongside the dominance of others [45,78]. Thus from the cohort of EFs in a given ecosystem, there may emerge a multitude of ‘EF groupings’ identifiable by similarities in (i) the main contributing group (trophic level or functional group), (ii) functional traits, and (iii) biodiversity mechanisms underpinning them.

More comprehensive study of diverse B–EF relationships must first be conducted before EF groupings may be confidently established. First, focus has been largely on EFs promoted by primary producers, and many animal-mediated EFs (excepting invertebrate pollinators and detritivores) are currently understudied: e.g. herbivory, seed dispersal or nutrient filtration [51]. At least for herbivorous [99] and seed-dispersing vertebrates [100], functional redundancy may be low; however, trait-based assessments of these B–EF relationships are rarely conducted (but see [101]). This is a vital future research area, as interactions across trophic levels are key to promoting EFs [49,54,55]. For example, the impact of herbivore diversity on grassland EFs may be substantial [68], and intensive herbivory may impact the strength of observed plant B–EF relationships [102]. Second, B–EF studies have still mostly considered single or a few EFs (in particular, e.g. NPP, biomass production; but see [69–71,81]), and few have explored the relative influences of functional diversity and dominant traits on multiple EFs. This should now be a research priority; considering individual EFs separately in multifunctionality studies (*sensu* [45]; see also [97]). Finally, trait-based B–EF studies have been conducted in few ecosystem types (mostly grasslands and forests). Thus, we currently have limited understanding of potential ecosystem controls on the importance of complementarity mechanisms (e.g. [44,45,80,87]). B–EF research must now look to further trait-based study of the mechanisms promoting multiple EFs across under-studied ecosystem types; in particular, highly species-rich systems ([84,85,90]; see also [44] for an important framework).

#### (ii) Comparing EF portfolios with multiple EF groupings to examine EF trade-offs

Exploring potential trade-offs in underlying EFs enables us to better understand mechanistic drivers of final ES and the trade-offs that may exist in their delivery. While recent work has illustrated trait-based pathways to underlying EF trade-offs [20], to date there does not exist a framework which (i) incorporates multiple mechanistic B–EF relationships from multiple trophic levels (but see [49]), (ii) can account for B–EF relationships from multiple ecosystem components [27] and (iii) can contrast these B–EF relationships across pairings of final ES and ecosystem types [18]. We propose that overlapping EF portfolios with EF groupings identified through future B–EF research provides a rudimentary means to assess final ES trade-offs through the full mechanistic pathway.

**Figure 3.**
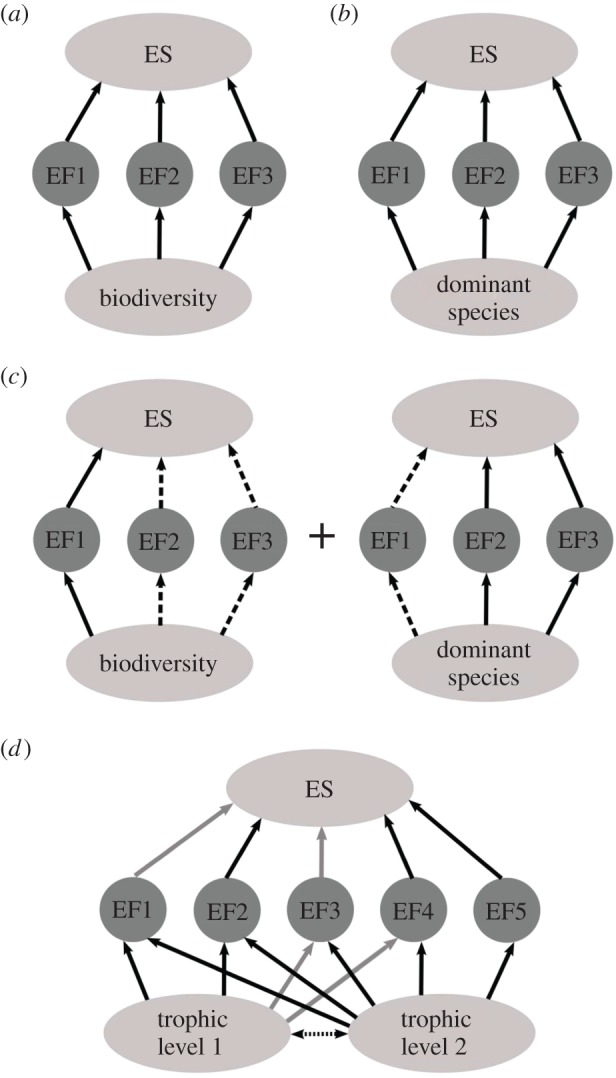
Hypothetical variation in B–EF–ES relationships (see also [18]) as driven by the main contributing EFs (within an EF portfolio). Black arrows refer to positive effects (dashed arrows displaying less strong effects) while grey arrows refer to negative effects.

In comparing EF portfolios with general EF groupings in a specific context, a number of scenarios may emerge. First, the EF portfolio for a final ES may be predominantly promoted by one EF grouping (all key EFs promoted by the same functional traits via the same main mechanism). Depending on the mechanism driving the EF grouping (diversity or dominance [44]), we can determine a strong positive (figure 3*a*) or a negligible influence (figure 3*b*) of biodiversity on final ES delivery in a given context (e.g. agricultural pollination [15,52,53]; timber production [16,71]). Second, an EF portfolio may be promoted by multiple EF groupings according to (i) different mechanisms and (ii) different functional traits of the main contributing group of organisms: e.g. potential EF trade-offs under different biodiversity scenarios [20]. For example, dominance of high root : shoot ratio and height traits (high rooting [103] and vertical biomass production) and diversity in growth form traits (above-ground structural variation) in vegetation communities may contribute positively to coastal storm attenuation (figure 3*c*). Here high species richness and distinctive traits of rare species [78] may provide additional complementarity to driving key EFs [44,69,70,73,74]. Finally, multiple EF groupings may comprise the EF portfolio, promoted by various trophic levels (both positively and negatively): e.g. final ES (i) delivered via EFs pertaining to multiple ecosystem compartments (e.g. carbon storage [29,71]; see also [27]; box 2), or (ii) controlled strongly by multi-trophic interactions (e.g. timber or fodder production [44,68]). A plethora of contributing EFs and strong species interactions may mean high levels of biodiversity drive final ES (figure 3*d*; [85]), and further work is required to understand relative trophic controls [49].

Overlapping EF portfolios and corresponding EF groupings may further enable us to establish mechanistic drivers of trade-offs between final ES. Via simple comparison of the overlap between the EF portfolios of two final ES and the EF groupings encompassing them, we may begin to identify differences in the traits and mechanisms chiefly promoting them in given contexts. Rigorous continued multiple B–EF research across a wide range of ecosystem types will further enable comparison of how and when the traits and biodiversity mechanisms promoting them result in synergies and trade-offs in final ES delivery across contexts. Such an approach may vastly improve current predictability of ES synergies and trade-offs, and future findings may be compared with those from ES bundles research [15,19,94] to elucidate mechanistic underpinnings of observed B–ES relationships in space.

## 5. Conclusion

Over the last decades, we have seen substantial research quantifying biodiversity's role in promoting EFs and ES [5,6,68,93]; we are rapidly gaining insight into (i) the mechanisms by which organisms promote different EFs [44–46,85], (ii) the tendency for synergies and trade-offs between ES across landscapes [15,19,94] and (iii) how scenarios of management and land-use change interact with these associations [104]. Conceptual frameworks are emerging mechanistically linking multiple facets of biodiversity to ES delivery [14,20,44,49], and the vulnerability of specific ES to biodiversity loss via these functional linkages [14,49,105]. However, what is lacking is a means to bring all of these avenues together to understand and predict the ES impacts of biodiversity change. Vital research avenues to work towards this goal lie in (i) improving coverage of EFs, higher trophic levels and understudied ecosystems in observational B–EF research, (ii) working to identify generalities in the traits and mechanisms involved in multiple B–EF relationships, (iii) moving from proxies to considering final ES as the net product of key underpinning EFs (EF portfolios), (iv) identifying trait-based synergies and trade-offs between EFs and how these extend to final ES trade-offs and finally (v) exploring context-dependency to these mechanisms and associations (and their implications for landscape management [106]). The road ahead to establishing these goals is long and data-intensive, but the outlook is that we may already possess many of the tools required to reach a greater mechanistic understanding and predictability of B–ES relationships.
